# The Exoscope in Neurosurgery: An Overview of the Current Literature of Intraoperative Use in Brain and Spine Surgery

**DOI:** 10.3390/jcm11010223

**Published:** 2021-12-31

**Authors:** Nicola Montemurro, Alba Scerrati, Luca Ricciardi, Gianluca Trevisi

**Affiliations:** 1 Department of Neurosurgery, Azienda Ospedaliera Universitaria Pisana (AOUP), University of Pisa, 56100 Pisa, Italy; 2 Department of Neurosurgery, Sant’Anna University Hospital, 44124 Ferrara, Italy; scrlba@unife.it; 3 Department of NESMOS, Neurosurgery, Sant’Andrea Hospital, “Sapienza” University of Rome, 00189 Rome, Italy; ricciardi.lu@gmail.com; 4 Department of Neurosurgery, Presidio Ospedaliero Santo Spirito, 65124 Pescara, Italy; trevisi.gianluca@gmail.com

**Keywords:** exoscope, high-definition 3D exoscope, augmented reality, intraoperative visualization, neurosurgery, brain tumor, virtual reality, spine, neuronavigation

## Abstract

Background: Exoscopes are a safe and effective alternative or adjunct to the existing binocular surgical microscope for brain tumor, skull base surgery, aneurysm clipping and both cervical and lumbar complex spine surgery that probably will open a new era in the field of new tools and techniques in neurosurgery. Methods: A Pubmed and Ovid EMBASE search was performed to identify papers that include surgical experiences with the exoscope in neurosurgery. PRISMA guidelines (Preferred Reporting Items for Systematic Reviews and Meta-analyses) were followed. Results: A total of 86 articles and 1711 cases were included and analyzed in this review. Among 86 papers included in this review 74 (86%) were published in the last 5 years. Out of 1711 surgical procedures, 1534 (89.6%) were performed in the operative room, whereas 177 (10.9%) were performed in the laboratory on cadavers. In more detail, 1251 (72.7%) were reported as brain surgeries, whereas 274 (16%) and 9 (0.5%) were reported as spine and peripheral nerve surgeries, respectively. Considering only the clinical series (40 studies and 1328 patients), the overall surgical complication rate was 2.6% during the use of the exoscope. These patients experienced complication profiles similar to those that underwent the same treatments with the OM. The overall switch incidence rate from exoscope to OM during surgery was 5.8%. Conclusions: The exoscope seems to be a safe alternative compared to an operative microscope for the most common brain and spinal procedures, with several advantages that have been reached, such as an easier simplicity of use and a better 3D vision and magnification of the surgical field. Moreover, it offers the opportunity of better interaction with other members of the surgical staff. All these points set the first step for subsequent and short-term changes in the field of neurosurgery and offer new educational possibilities for young neurosurgery and medical students.

## 1. Introduction

The surgical microscope has represented a basic tool in neurosurgery since the late 1960s, and it continues to be critically essential in the microsurgical treatment of brain and spine pathologies [1,2,3,4,5,6,7,8]. Advances in digital imaging, WiFi internet connections, screen technology and optics have led to the development of extracorporeal telescopes, also known as exoscopes, which represent valuable alternatives to traditional OMs for surgical magnification [6,9,10]. The use of the microscope requires that surgeons look directly through the surgical microscopic objective lenses to visualize the target area; however, it seems that this “face-to-machine” interface has been overcome due to the introduction and use of new digital 3-dimensional (3D) imaging exoscopes [2].

As widely reported in microsurgery and minimally invasive procedures, the pursuit of highly detailed images and techniques has been providing both valuable clinical results and patient satisfaction [11]. The technology of exoscopes has continuously changed over the years, and these devices are often updated in their software and hardware. Exoscopes have been the latest addition to the neurosurgeons’ armamentarium, acting as a bridge between OMs and endoscopes [12]. The development of the 3D exoscope represents a marvel of technological innovation in modern surgical practice, which continues to renew itself year by year, from the first 3D High Definition (HD) visualization exoscope to the most recent 3D 4K exoscope. Furthermore, these modern exoscopes are embedded with light filters for 5-aminolevulinic acid (5-ALA) and indocyanine video-angiography, pneumatic arms, adjustable operative settings, multiscreen output, longer focus distance, and greater magnification powers [13,14].

3D exoscopes are novel high-definition digital camera systems that are able to deliver intense light and magnification to the deepest areas of the surgical field, allowing the surgeon to see, through 3D glasses and a 3D monitor, critical neural and vascular structures as well as tissue differentiation with high magnification. A surgeon’s position is not limited to the microscope’ oculars, while freedom in movements during surgery, a higher comfort rate, a lower fatigue after longer procedures have been already reported in using an exoscope [9,15,16,17,18]. In neurosurgery, supports for various digitized information are essential for improving operative grades, as neurosurgeons could benefit from a new surgeon’s eye that visualizes the operative field with integration of others medical information [10,17].

Exoscopes, as well as other modern devices, require specific training, although the learning curve is very short when compared to other neurosurgical systems such as operative microscopes (OM) and endoscopes [5,6,7,19]. Siller et al. [5] reported no significant differences among patients who underwent surgery with OM or exoscope for lumbar posterior decompression (LPD) and anterior cervical discectomy and fusion (ACDF). Similarly, Muhammad et al. [7] reported results in cranial surgery comparable to the OM with better visual quality and greater comfort for the surgeon. The exoscope system is a safe and effective alternative or adjunct to the existing binocular OM for brain tumor, skull base surgery, aneurysm clipping and vascular microanastomosis, both cervical and lumbar complex spine surgery [5,6,7,8,10,20,21,22,23,24,25]. The exoscope provides the surgeon with a comfortable, high-resolution visualization without compromising surgical exposure and patient safety. The integrated features like the lock-on-target and waypoints together with the footswitch allow the surgeon to efficiently place the camera and to return to saved positions, even hands-free. All these functions in combination with the digital visualization are convenient and ergonomic compared to OM, even when the surgeon has to see into the situs using extreme angles. To date, several exoscopic systems are available for neurosurgical use. VITOM^®^ (Karl Storz, Tuttlingen, Germany), ORBEYE™ (Olympus, Tokyo, Japan), Modus V™ (Synaptive Medical, Toronto, ON, Canada), Kinevo 900 (Carl Zeiss Meditec AG, Jena, Germany), BrainPath^®^ (Nico Corporation, Indianapolis, IN, US) and Aeos^®^ (Aesculap, Tüttlingen, Germany) are the most commonly used, with different technical, software and hardware characteristics, but with the same goal [7,15,26,27,28,29,30]. As the exoscope will probably open a new era in the field of new tools and techniques in neurosurgery, as the OM did in the 1960s, this review aims to investigate about the use of the exoscope in preclinical and clinical neurosurgical settings, the most common neurosurgical procedures performed with the exoscope, as well as the impact of exoscope on surgical outcome and workflow, reporting operative complications, surgical procedures switched from exoscope to OM, and advantages and disadvantages compared to the microscope.

## 2. Materials and Methods

### 2.1. Literature Search

A Pubmed and Ovid EMBASE search was performed to identify papers that include surgical experiences with the exoscope in neurosurgery. PRISMA guidelines (Preferred Reporting Items for Systematic Reviews and Meta-analyses) were followed [31]. The key words “exoscope”, “exoscopic visualization”, “neurosurgery”, “brain”, “spine” and “cadaver lab” were used in both “AND” and “OR” combinations. The key words and the detailed search strategy are reported in Table 1.

The inclusion criteria were the following: case series or case report reporting clinical data and neurosurgical intraoperative experiences with exoscope (both 2D and 3D visualization, as well 3D 4K definition) in brain and spine surgery as well as laboratory experiences in the field of neurosurgery. Exclusion criteria were the following: (1) studies published in languages other than English with no available English translations, (2) review articles, (3) studies with insufficient data, (4) studies not related with this topic.

### 2.2. Data Collection

From each study, we extracted the following data: (1) number of neurosurgical procedures performed using the exoscope divided by cerebral (tumor and vascular), spinal and peripheral nerve pathology as well as laboratory experiences; (2) exoscope manufacturer and/or model; (3) visualization mode setting; (4) operative complications and surgical procedures switched from the exoscope to OM; (5) advantages and disadvantages identified by authors (video image quality, surgical field, handling, surgical ergonomics, educational usefulness, depth perception, operative time and/or workflow, operative team involvement).

### 2.3. Outcomes

The primary objective of this review was to examine the most common neurosurgical procedures performed with the use of the exoscope and to identify surgical workflows, operative complications during the use of the exoscope and surgical procedures switched from the exoscope to the OM. The secondary objective was to report the most common advantages and disadvantages identified by authors (video image quality, surgical field, handling, surgical ergonomics, educational usefulness, depth perception, operative time and/or workflow, operative team involvement) to highlight strengths and weaknesses of this new technology.

## 3. Results

**Figure 1:**
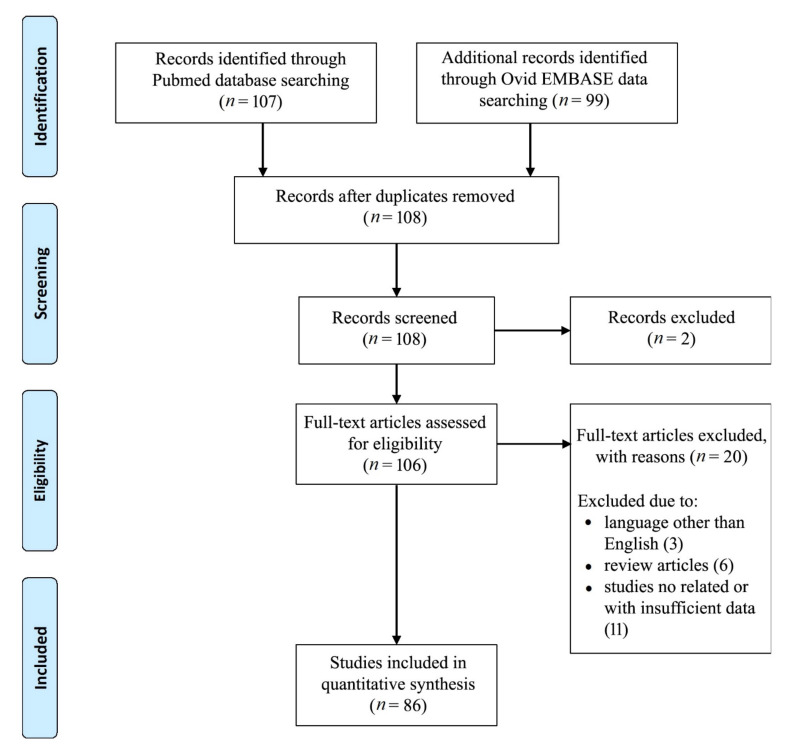
PRISMA flow diagram.

The database search yielded 208 articles. After the removal of duplicates, 108 articles were eligible for screening. A total of 86 articles met the selection criteria [3,4,5,6,7,8,9,10,13,15,16,19,21,23,25,26,27,28,29,30,32,33,34,35,36,37,38,39,40,41,42,43,44,45,46,47,48,49,50,51,52,53,54,55,56,57,58,59,60,61,62,63,64,65,66,67,68,69,70,71,72,73,74,75,76,77,78,79,80,81,82,83,84,85,86,87,88,89,90,91,92,93,94,95,96,97]. The search flow diagram is shown in Figure 1.

### 3.1. Demographic and Number of Neurosurgical Procedures Performed Using Exoscope 

Studies included in our review are summarized in Table 2. A total of 86 articles and 1711 cases were included and analyzed in this review [3,4,5,6,7,8,9,10,13,15,16,19,21,23,25,26,27,28,29,30,32,33,34,35,36,37,38,39,40,41,42,43,44,45,46,47,48,49,50,51,52,53,54,55,56,57,58,59,60,61,62,63,64,65,66,67,68,69,70,71,72,73,74,75,76,77,78,79,80,81,82,83,84,85,86,87,88,89,90,91,92,93,94,95,96,97]. Among 86 papers included in the review 74 (86%) were published in the last 5 years, showing an increasing interest in the use of the exoscope in the operating room in neurosurgery. Out of 1711 surgical procedures, 1534 (89.6%) were performed on human beings in the operative room, whereas 177 (10.9%) were performed in the laboratory on cadavers. A total of 1251 (72.7%) were reported as brain surgeries, whereas 274 (15.9%) and nine (0.5%) were reported as spine and peripheral nerve surgeries, respectively. From this review of the literature, more than 311 gliomas, 171 brain metastasis and 97 meningioma and 244 pituitary adenomas were resected by using the exoscope. One hundred intracerebral hemorrhage (ICH) and 24 neurovascular conflicts were treated by using the exoscope. In spine surgery, 64 cervical disease, 189 lumbar pathologies and 12 spine tumors were reported. A total of 48 papers (55.8%) reported their experience with a 3D HD exoscope, of which 15 used a K4 monitor, reporting the use of a 4K HD exoscope in 17.4% of the papers. Excluding the 10 papers that report laboratory experiences, 21 papers (27.6%) resulted in single case reports, 12 papers (15.8%) were small series (with ≤5 patients treated) and 43 papers (56.6%) were clinical studies with a mean of 32.8 patients and a median of 18 patients. Most common exoscope manufacturers and/or models resulted VITOM^®^ (Karl Storz, Tuttlingen, Germany) and ORBEYE^®^ (Olympus, Tokyo, Japan), which were used and reported in 36% and in 23.3% of these review papers, respectively. Modus V™ (Synaptive Medical, Toronto, ON, Canada), BrainPath^®^ (Nico Corporation, Indianapolis, IN, USA) and Kinevo 900 (Carl Zeiss Meditec AG, Jena, Germany) exoscopes were used in 9.3%, 7% and 3.5% of papers reported in this review. Table 2 and Table 3 show all details about cranial and spine/peripheral nerve surgical procedures. Table 4 shows laboratory experiences with an exoscope.

### 3.2. Evaluation of Exoscopic Surgical Procedures

Excluding case reports and considering clinical series reporting surgical complications, 40 studies and 1328 patients were assessed, reporting an overall surgical complication rate of 2.6% during the use of the exoscope. These patients experienced complication profiles similar to those that underwent the same treatments with the OM. Similarly, 21 clinical series with a total of 891 patients reported an overall switch incidence rate from exoscope to OM during surgery of 5.8% (52 cases). A total of 30 articles reported a qualitative comparison between the exoscope and the OM, while a total of 12 papers reported a quantitative, concrete and prospective comparison between one or more common features of the exoscope with the OM. The video image quality, 3D visualization and surgical filed with exoscopes were rated superior to similar to those of OMs in all papers. The comfort level of surgeon’s posture during surgery, the educational usefulness, and the operative team involvement with the exoscope were assessed as superior compared to OM. Otherwise, depth perception was rated to be similar or inferior to the OM. Workflow and operative time were evaluated as equal or slightly higher than those of OMs. Table 5 shows all details.

## 4. Discussion

There were over 1524 surgeries that reported using an exoscope: 1251 (72.7%) on brain, whereas 274 (15.9%) and 9 (0.5%) on spine and peripheral nerves, respectively. Among these, more than 311 were gliomas, 171 were brain metastasis and 97 were meningioma and 244 were pituitary adenoma, 100 resulted ICH and 24 were neurovascular conflicts. Similarly in spine, 64 cervical and 189 lumbar pathologies were treated with the use of exoscope in the operative room, as well as 12 spine tumors were reported.

The development of surgical magnification and neurosurgery progressed on separate paths until the 1960s, when the merging of these two innovations led to the rapid growth of cerebral surgery [98,99,100,101]. From that time, intraoperative technological advances improved, and the OM and endoscope allowed complete resection of glioma and other intraventricular and pituitary tumors, neurovascular and spine diseases, under magnification with good lighting and through minimally invasive approaches [12,15,16,45,47,57,58,60,95,102,103].

Neuronavigation, ultrasound, intraoperative magnetic resonance imaging (MRI) and/or computed tomography (CT) scan, robotic technology, augmented reality and awake surgery increased the ability of the neurosurgeon to perform safe and maximal tumor resection [12,102,104,105,106,107,108,109,110]. Exoscopes launched a new era in the field of neurosurgery. These exoscopes are designed to provide high-resolution 3D imaging of the structure of tissue, blood vessels and other features to enable more accurate surgery and, including a display video, allow for simultaneous surgical team viewing. Exoscopes represent the next generation of operative imaging, helping the neurosurgeon to operate in a more ergonomic sitting position, facilitating the surgery team and reducing surgeon fatigue by reducing the amount of time practitioners would have to view the images through a microscope eyepiece. These systems work to bridge the gap between OM and endoscopes by combining the form factor of the endoscope with the image quality of the microscope [43,49,78,94]. Some disadvantages of exoscopic visualization were reported, especially in the early 2D exoscope, such as a limited applicability in deep seated cranial pathologies and tissue identification in case of bleeding, a magnification of deep-seated pathologies and above all the lack of stereopsis. All of these disadvantages seem to be solved with new 3D exoscopes, which however led to headache and nausea in very few cases due to the use of polarized glasses [19,41,51,52]. Furthermore, these devices still have usage limitations due to their high cost and to the impossibility, at the moment, to use 5-ALA fluorescence for tumor resection. A major advantage of the exoscope is the shared 3D view for all participants in the procedure [28,67,95]. The possibility to look at the same time in the same monitor allows more than one surgeon to operate and improves efficiency by sharing information with all surgical staff. Although Takahashi and colleagues [61] reported that assistant surgeons could sometimes experience a rotated view of the monitor; in this case the use of two or more 3D monitors in the operative room can solve this problem.

One of the characteristics ascribed to exoscopes is that they are superior to a conventional OM in terms of ergonomic features both in brain and spine surgery [37,39,49,52,67,69,90,91,92,94,95,96,97,111,112,113,114,115], as the ergonomic handling and the ease of intraoperative positioning of the device were found to be beneficial. Second, 3D monitors lead to an improved involvement of the co-surgeon and the scrub nurse during the procedure, and although some authors were satisfied with the high-resolution 3D digital images during surgery [52,60,68,73], others were not satisfied with the visual quality. In spine surgery, when two neurosurgeons are operating facing each other, the use of 2 monitors each positioned in front of each surgeon allows extreme freedom of movement and modification of the surgical corridor [89]. The important aspect of the exoscope monitor is that the surgeon, assistant and nurse all see the same image with the same quality and the exoscope does not interfere with communication and allows all surgical staff to feel more involved in the surgical procedure [7]. By increasing the visualization of anatomic details helps to identify the different layers and the tumor-nerve interface, and exoscopes can be useful also for peripheral nerve sheath tumors to preserve functional fascicles achieving gross-total resection [88].

Exoscopic tools seem to shift from cortical cranial tumor surgery to deep-seated brain tumors, as exoscope technology has progressively improved during the last few years, with results in terms of clinical outcome and surgical complications similar to conventional OM [85]. Hafez and colleagues [23] reported the largest comparative and laboratory series with the use of the exoscope and OM and showed that both methods are effective in doing bypass suturing, whereas the suturing time was less using the microscope and stitch distribution was better using the exoscope. Among brain tumors, Gonen and colleagues [45] reported the largest series of glioma resection (56 patients) using the exoscope, accounting for 44 cases of high-grade gliomas and 12 of low-grade gliomas and reporting just one (1.8%) perioperative complication (hemorrhage within the resection bed) in a patient with glioblastoma multiforme. Similarly, Gassie et al. [55], Piquer et al. [39], Day [44] and Eichberg et al. [30] reported that 30, 25, 22 and 12 patients, respectively, underwent surgical resection for glioma using different exoscopes. Overall postoperative surgical complications with permanent motor deficit range from 0% to 8% [30,39,44,55]. Rotermund et al. [95] reported the largest series of patients underwent transsphenoidal surgery for pituitary adenoma (239 patients), reporting that no serious episodes or minor complications occurred based on the usage of the exoscope, as well as no significant differences regarding the duration of surgery, complications or extent of resection compared to conventional microscopy. Chen et al. [73] reported a total of 81 patients received tumor resection through the retrosigmoid approach with either an exoscope (39 patients) or an OM (42 patients). Patients in the two groups had comparable tumor location (*p* = 0.439) and Koos grading (*p* = 0.867). There were significant differences in the duration of surgery (*p* = 0.172), the extent of tumor resection (*p* = 0.858), facial function (*p* = 0.838) and hearing ability (*p* = 1.000). Gonen et al. [45], Khalessi et al. [58], Ahmad et al. [9] had a total of 35 patients with neurovascular pathologies (aneurysms, arteriovenous malformations, cavernomas) who underwent surgery with an exoscope, reporting an overall good outcome and only 2.8% postoperative complications. In particular, Ahmad et al. [9] reported 12 microvascular anastomosis, reporting no difference in operative time (*p* = 0.714), ischemia time (*p* = 0.972), or microsurgical complications (*p* = 1) between the ORBEYE and conventional microscopy groups.

Regarding 3D visualization, Ricciardi et al. [14] in a previous review comparing exoscopes and microscopes found that image quality, optical power and magnification of the exoscope were rated at least equivalent to the microscope. In addition, exoscopes are also able to allow the surgeons to quickly switch from a micro to a macro vision and vice versa, when necessary, to explore all corners of the surgical field and to keep an eye on any bleeding [35,39,52]. Nevertheless, at present exoscopes have some limitations. Burkhardt et al. [71] reported that in 5 out of 10 cases (50%) of cranial surgery, a switch to the OM was necessary, due to the need to use 5-ALA fluorescence guided visualization in two cases and because the illumination of the depth of the operative field was not sufficient in 3 cases. Lin et al. [81] obtained gross total resection in all four cases of intraventricular meningiomas, reporting no intraoperative complications nor conversion to microscopic or open approach. Ridge et al. [116] and Teo et al. [29] highlighted the role of the exoscope in reducing the risk of infection exposure to the surgical team during the COVID-19 pandemic [117,118,119].

The use of the exoscope has been largely reported with a variety of different exoscope models used also in spinal surgery [5,7,15,26,29,41,71,89]. Ariffin et al. [15] submitted an interesting series of minor to major surgical spine procedures in 69 patients using the exoscope, reporting only four cases (5.8%) of dural tear as surgical complications and no postoperative neurological deficits. Similarly, Siller et al. [5] (40 patients undergoing lumbar posterior decompression (LPD) and 20 patients undergoing anterior cervical discectomy and fusion (ACDF), showing no intraoperative complications by using the exoscope, and reported similar results in outcome compared to controls in whom an OM was used. According to the attending surgeon, the intraoperative handling of the instruments was rated to be comparable to that of the OM, while the comfort level of the surgeon’s posture intraoperatively (especially during “undercutting” procedures) was rated as superior [15]. Otherwise, Burkhardt et al. [71], including 16 cranial and 18 spinal surgical procedures in their paper, reported some intraoperative difficulties and that one spinal and five cranial procedures switched to OM or the endoscope for the following reasons: poor illumination (four cases), tissue identification (one case), and the need for fluorescence imaging (one case).

This review shows how a such large number of published papers and patients underwent brain and spinal surgery with the exoscope, showing the simplicity of use, the total safety for the patient, the good 3D vision and magnification of the surgical field and the opportunity of better interaction with other members of the surgical staff. All these points set the first step for subsequent and short-term changes in the field of neurosurgery and offer new educational possibilities for young neurosurgery and medical students. This review has some limitations. First, this review is susceptible to changes over the short term, as exoscopes were increasingly used in recent years and therefore an increasing number of papers will be published in the near future, and because technology and science advance incessantly. Second, this review aims to show the advantages and disadvantages of a new tool used in neurosurgery, reporting surgical experiences of different authors and summarizing the current literature, without drawing unique technical conclusions, as we believe it is still too early at this moment. Future clinical studies and reviews are needed to demonstrate if exoscopes will change the neurosurgical sciences.

## 5. Conclusions

Exoscopes have been used constantly in an increasing number of surgical procedures all around the world, suggesting that they could ultimately replace the OM in the future and represent the beginning of a new era of intraoperative visualization in neurosurgery. A 3D exoscope seems to be a safe alternative compared to the OM for most common brain and spinal procedures, with several advantages that have been reached. This review confirmed the role of the exoscope as a new tool that can help surgeons during surgery and even replace the OM in the near future due to several aspects: a better ergonomic posture of the surgeons during surgical procedures, the possibility to improve neurosurgical education, and in creating a better and effective operational team involvement. The quality of images and 3D 4K videos in most recent exoscopes has been increasingly improved in recent years, although at the moment the most reported drawback remains the slight lack of depth perception. The exoscope itself can be considered a useful educational tool in neurosurgery. As with other adaptations of new technology, it will take some time for systems to be tweaked and the pros and cons of different approaches to be better appreciated. More research needs to be done. A short learning curve is mandatory.

## Figures and Tables

**Table 1:** Search syntax.

PubMed Search Accessed on 5 July 2021 (108 Articles)	Embase Search Accessed on 5 July 2021 (106 Articles)
(exoscope OR exoscopic visualization) AND (neurosurgery OR brain OR spine OR cadaver lab)	(‘exoscope’ OR ‘exoscopic visualization’) AND (‘neurosurgery’ OR ‘brain’ OR ‘spine’ OR ‘cadaver lab’)

**Table 2:** Summary of cranial studies included in the review.

Authors	Year	Neurosurgical Procedures		Total	Exoscope Manufacturer and/or Model	Visualization Mode Setting
		Tumor (*n*°)	Vascular and Others Disease (n°)			
Gildenberg & Labuz [32]	1997	glioma (17), metastasis (1)	-	18	N/A	-
Mamelak et al. [34]	2010	glioma (3), HMG (1), meningioma (3), LGG (1), pituitary adenoma (1)	vagus nerve stimulator (1)	10	HDXO-SCOPE, Karl Storz	HD 2D
Mamelak et al. [36]	2012	germinoma (1)	-	1	VITOM^®^	HD 2D
Belloch et al. [37]	2014	GBM (15), AA (2), metastasis (3), LGG (3)	-	23	HDXO-SCOPE, Karl Storz	HD 2D
Birch et al. [38]	2014	pineocytoma (3), germinoma (1), lipoma (1)	-	5	HDXO-SCOPE, Karl Storz	HD 2D
Piquer et al. [39]	2014	GBM (23), AA (2), metastasis (3), LGG (2)	-	30	VITOM^®^	HD 2D
Ritsma et al. [40]	2014	-	ICH (1)	1	Mi SPACE	HD 2D
Parihar et al. [41]	2016	meningioma (5), glioma (4), HMG (1), metastasis (1), schwannoma (3), neurocytoma (1), medulloblastoma (1), craniopharyngioma (1)	ICH (3), colloid cyst (1), arachnoid cyst (1), abscess (2), trigeminal neuralgia (1)	25	VITOM^®^	HD 2D
Scranton et al. [42]	2016	-	cavernoma (2)	2	N/A	HD 2D
Bauer et al. [43]	2017	-	ICH (18)	18	BrainPath^®^	HD 2D
Day [44]	2017	GBM (15), AA (4), ependymoma (2), neurocytoma (1), LGG (1), metastasis (20)	ICH (6)	49	BrainPath^®^	HD 2D
Gonen et al. [45]	2017	astrocytoma (56), meningioma (40), metastasis (33), schwannoma (5), epidermoid/dermoid cyst (3), paraganglioma (2), craniopharyngioma (1), pituitary adenoma (1), miscellaneous (8)	aneurysms (7), AVM (5), dAVF (1), ICH (20), trigeminal neuralgia (7), hemifacial spasm (1), arachnoid cysts (2), Chiari I (1), infection (3), colloid cyst (4)	200	ROVOT-m	HD 2D
Jackson et al. [46]	2017	GBM (3), AA (3), metastasis (1), lymphoma (2)	demyelinating disease (2)	11	VITOM^®^	HD 2D
Krishnan et al. [47]	2017	-	anastomosis (3), AVF (1), ICH (1)	3	VITOM^®^	HD 2D
Labib et al. [48]	2017	-	ICH (39)	39	Mi SPACE	HD 2D
Nagata et al. [50]	2017	-	hemifacial spasm (2)	2	ORBEYE^®^	HD 2D
Oertel & Burkhardt [51]	2017	metastasis (3), lymphoma (1)	trigeminal neuralgia (1)	5	VITOM^®^	HD 3D
Rossini et al. [52]	2017	meningioma (1)	-	1	VITOM^®^	HD 3D
Weiner & Placantonakis [53]	2017	JPA (1)	-	1	VITOM^®^	HD 3D
Beez et al. [54]	2018	astrocytoma (2)	myelomeningocele closure (1)	3	VITOM^®^	HD 3D
Gassie et al. [55]	2018	GBM (24), AA (6), metastasis (14), lymphoma (2)	cavernoma (2), demyelinating disease (2)	50	VITOM^®^	HD 2D
Griessenauer et al. [56]	2018	-	ICH (5)	5	BrainPath^®^	HD 2D
Iyer & Chaichana [57]	2018	GBM (11), AA (3)	-	14	VITOM^®^	HD 2D
Khalessi et al. [58]	2018	meningioma (1), glioma (1)	clipping (4), cavernoma (3), AVM (2), endarterectomy (1), CSDH (1), cyst (1), Chiari I (1)	17	ORBEYE^®^	HD 3D
Klinger et al. [59]	2018	-	aneurysm (1)	1	Modus V™	HD 3D
Mampre et al. [60]	2018	metastasis (11), HMG (2)	cavernoma (2)	15	VITOM^®^	HD 2D
Sindelar et al. [8]	2018	-	ICH (1)	1	BrainPath^®^	HD 2D
Takahashi et al. [61]	2018	meningioma (5), pituitary adenoma (1), GBM (1), HMG (2), metastasis (1), craniopharyngioma (1)	Moyamoya disease (2), congenital dermal sinus (1)	14	ORBEYE^®^	HD 2D
Akbari et al. [62]	2019	metastasis (4), GBM (3), LGG (2), AA (1)	-	10	VITOM^®^	HD 2D
Angileri et al. [4]	2019	-	cavernoma + HMG (1)	1	VITOM^®^	HD 3D
Bakhsheshian et al. [63]	2019	metastasis (25)	-	25	BrainPath^®^	HD 3D
Garneau et al. [16]	2019	schwannoma (4)	temporal lobe encephalocele (2)	6	Modus V™	HD 2D
Li Ching Ng & Di Ieva [66]	2019	-	MVD (1)	1	VITOM^®^	HD 3D
Muhammad et al. [7]	2019	schwannoma (1), meningioma (3)	-	4	Modus V™	HD 3D
Murai et al. [67]	2019	meningioma (3), schwannoma (3), pituitary adenoma (1), GBM (1)	clipping (3), bypass (2), carotid endarterectomy (2), ICH (3)	18	ORBEYE^®^	3D 4K
Nossek et al. [68]	2019	-	bypass (5)	5	ORBEYE^®^	3D 4K
Smith et al. [69]	2019	-	skull base (11)	11	ORBEYE^®^ (10), VITOM^®^ (1)	HD 3D
Ahmad et al. [9]	2020	-	microvascular anastomosis (12)	12	ORBEYE^®^	HD 3D
Baron et al. [10]	2020	GBM (28)	-	28	Modus V™	HD 3D
Burkhardt et al. [71]	2020	metastasis (3), LGG (1), AA (1), GBM (2), meningioma (1), subependymoma (1), lymphoma (1)	cavernoma (1), ICH (1), aneurysm (2), CSF leak (1), trigeminal neuralgia (1)	16	VITOM^®^	HD 3D
Chakravarthi et al. [72]	2020	hypothalamic mass (1)	-	1	Kinevo 900	HD 3D
Chen et al. [73]	2020	schwannoma (39)	-	39	VITOM^®^	HD 2D
Doglietto et al. [76]	2020	GBM (1)	-	1	ORBEYE^®^	3D 4K
Eichberg et al. [30]	2020	GBM (13), metastasis (19), glioma (8)	cavernoma (7), colloid cyst (4), other (5)	56	BrainPath^®^	HD 2D
Fuse et al. [3]	2020	meningioma (1)	-	1	VITOM^®^	HD 2D
Garneau et al. [77]	2020	-	temporal lobe encephalocele (1)	1	Modus V™	HD 3D
Khatri et al. [78]	2020	craniopharyngioma (1)	-	1	N/A	-
Kleshchova et al. [79]	2020	endodermal cyst (1)	-	1	N/A	-
Ligas et al. [80]	2020	-	hemifacial spasm (1)	1	N/A	-
Lin et al. [81]	2020	meningioma (4)	-	4	VITOM^®^	HD 3D
Patel et al. [84]	2020	-	bypass (1)	1	N/A	-
Roethe et al. [85]	2020	GBM (9), meningioma (6), LGG (4), metastasis (3), AA (3)	cavernoma (1), trigeminal neuralgia (1), CSF fistula (1)	28	Kinevo 900	3D 4K
Silverstein et al. [87]	2020	-	aneurysm (1)	1	ORBEYE^®^	HD 3D
Amoo et al. [90]	2021	metastasis (5), meningioma (4), GBM (5), schwannoma (1), craniopharyngioma (1)	AVM (1), hemifacial spasm (1)	18	ORBEYE^®^	3D 4K
Marenco-Hillembrand et al. [92]	2021	metastasis (8), LGG (4), GBM (3)	-	15	N/A	-
Muscas et al. [27]	2021	meningioma (4), cranial nerve tumors (2), glioma (3), choroid plexus papilloma (1)	aneurysm (1), colloid cyst (1), neurovascular conflict (1), ethmoidal fistula (1)	14	ORBEYE^®^	3D 4K
Muto et al. [93]	2021	metastasis (5)	-	5	VS3 Iridium	HD 3D
Rennert et al. [94]	2021	GBM (1)	ICH (1)	2	VITOM^®^	HD 3D
Rösler et al. [26]	2021	GBM (6), pituitary adenoma (1), meningioma (1), craniopharyngioma (1), LGG (4), lymphoma (1), metastasis (3), HMG (1), hemangioma (1)	ICH (1), epilepsy (6), trigeminal neuralgia (1)	27	ORBEYE^®^	3D 4K
Rotermund et al. [95]	2021	pituitary adenoma (239), craniopharyngioma (12), meningioma (7), chordoma (4), metastasis (2)	other (32)	296	ORBEYE^®^	3D 4K
Shimizu et al. [96]	2021	meningioma (5), schwannoma (4)	trigeminal neuralgia (2), hemifacial spasm (3)	14	ORBEYE^®^	3D 4K
Strickland et al. [97]	2021	-	AVM (1)	1	N/A	-
Wali et al. [21]	2021	-	aneurysm (1)	1	ORBEYE^®^	3D 4K
Yoon et al. [25]	2021	metastasis (3), meningioma (3), GBM (4), HMG (1)	-	11	VOMS-100 (5), VITOM^®^ (6)	3D 4K

2d, 2 dimensional; 3D 4K, 3 dimensional 4K high-definition; AA, anaplastic astrocytoma; AVM, arteriovenous malformation; CSF, cerebrospinal fluid; CSDH, chronic subdural hematoma; dAVF, arteriovenous fistula; GBM, glioblastoma multiforme; HD, high definition; HMG, hemangioblastomas; ICH, intracerebral hemorrhage; N/A, not available; JPA, juvenile pilocytic astrocytoma; LGG, low-grade glioma; MVD, microvascular decompression.

**Table 3:** Summary of spine/peripheral nerve studies included in the review.

Authors	Year	Neurosurgical Procedures		Total	Exoscope Manufacturer and/or Model	Visualization Mode Setting
		Spine (n°)	Peripheral (n°)			
Mamelak et al. [34]	2010	ACDF (2), epidural abscess (1), lumbar discectomy (3)	-	6	HDXO-SCOPE, Karl Storz	HD 2D
Shirzadi et al. [19]	2012	LPD (11), TLIF (13)	-	24	VITOM^®^	HD 2D
Parihar et al. [41]	2016	neurofibroma (4), meningioma (1), ACDF (4), corpectomy (2), tuberculosis (1), lumbar discectomy (2)	-	14	VITOM^®^	HD 2D
Krishnan et al. [47]	2017	LPD (7), cervical foraminotomy (2), ACDF (1)	schwannoma (2), microneurorrhaphy (1)	13	VITOM^®^	HD 2D
Oertel & Burkhardt [51]	2017	ACDF (2), cervical laminectomies (2), TLIF (2), extradural tumor (1), LPD (1), lumbar discectomy (3)	-	11	VITOM^®^	HD 3D
Khalessi et al. [58]	2018	ACDF (1), disc herniation (2)	-	3	ORBEYE^®^	HD 3D
De Divitiis et al. [6]	2019	tumor (5)	-	5	VITOM^®^	HD 3D
Kwan et al. [65]	2019	ACDF (4), cervical corpectomy (1), cervical laminectomies (3), LPD (2)	-	10	ORBEYE^®^	HD 3D
Muhammad et al. [7]	2019	CPD (1), ACDF (1), disc herniation (2)	-	4	Modus V™	HD 3D
Murai et al. [67]	2019	LDP (3)	neurolysis (1)	4	ORBEYE^®^	3D 4K
Ariffin et al. [15]	2020	decompression (18), discectomy (17), TLIF (28), OLIF (6)	-	69	Kinevo 900	3D 4K
Barbagallo & Certo [70]	2020	ACDF (2)	-	2	VITOM^®^	HD 3D
Burkhardt et al. [71]	2020	ACDF (4), cervical laminectomies (1), metastasis (1), lumbar decompression (4), TLIF (1), disc herniation (5), dAVF (1), angiolipoma (1)	-	18	VITOM^®^	HD 3D
D’Ercole et al. [75]	2020	ALIF (9)	-	9	VITOM^®^	HD 3D
Oren et al. [82]	2020	disc herniation (1)	-	1	ORBEYE^®^	3D 4K
Roethe et al. [85]	2020	LPD (1)	-	1	Kinevo 900	3D 4K
Siller et al. [5]	2020	LDP (40), ACDF (20)	-	60	VITOM^®^	HD 3D
Teo et al. [29]	2020	fracture (2), meningioma (1), disc herniation (5)	-	8	Modus V™	HD 3D
Vetrano et al. [88]	2020	-	schwannoma (2)	2	ORBEYE^®^	3D 4K
Visocchi et al. [89]	2020	CVJ pathologies (6)	-	6	VITOM^®^ (3), ORBEYE^®^ (3)	3D 4K
Kim et al. [91]	2021	disc herniation (1)	-	1	N/A	-
Rösler et al. [26]	2021	ACDF (1), metastasis (1), tumor (1), schwannoma (2), LPD (4)	schwannoma (2), peripheral nerve (1)	12	ORBEYE^®^	3D 4K

3D 4K, 3 dimensional 4K high-definition; ACDF, anterior cervical discectomy and fusion; ALIF, anterior lumbar interbody fusion; CPD, cervical posterior decompression; CVJ, craniovertebral junction; HD, high definition; LPD, lumbar posterior decompression; OLIF, oblique lateral interbody fusion; TLIF, transforaminal lumbar interbody fusion.

**Table 4:** Summary of laboratory studies included in the review.

Authors	Year	Laboratory Neurosurgical Procedures (n°)	Total	Exoscope Manufacturer and/or Model	Visualization Mode Setting
Mamelak et al. [33]	2008	craniotomy (4)	4	HDXO-SCOPE, Karl Storz	HD 2D
Di Ieva et al. [35]	2012	suboccipital approach (20)	20	VITOM^®^	HD 2D
Moisi et al. [49]	2017	craniotomy (6)	6	Modus V™	HD 2D
Sack et al. [13]	2018	craniotomy (5)	5	ORBEYE^®^	HD 3D
Herlan et al. [64]	2019	pterional approach (6)	6	FA Aesculap	HD 3D
Crosetti et al. [74]	2020	dissection (4)	4	VITOM^®^	HD 3D
Hafez et al. [23]	2020	bypass anastomosis (100)	100	VITOM^®^	HD 3D
Pafitanis et al. [83]	2020	micro sutures (10), anastomoses (5)	15	Modus V™	HD 3D
Rubini et al. [86]	2020	skull base (12)	12	VITOM^®^	HD 3D
Hafez et al. [28]	2021	bypass (5)	5	AEOS	3D 4K

3D 4K, 3 dimensional 4K high-definition; HD, high definition.

**Table 5:** Summary of evaluation exoscopic surgical procedures studies.

Authors	Year	Patients (n°)	Operative complications (%)	Surgical Procedures Switched from Exoscope to OM (%)	Video Image Quality	Surgical Field	Handling	Surgical Ergonomics	Educational Usefulness	Depth Perception	Operative Time and/or Workflow	Operative Team Involvement
Mamelak et al. [34]	2010	16	0	Nd	+	+		+	+			+
Shirzadi et al. [19]	2012	24	0	Nd	=						=	
Belloch et al. [37]	2014	23	0	Nd			+	+				
Birch et al. [38]	2014	5	20	Nd		=					=	
Piquer et al. [39]	2014	30	0	Nd	+	+		+				
Parihar et al. [41]	2016	39	0	0	=			+		−		
Bauer et al. [43]	2017	18	0	Nd		=	=					
Day [44]	2017	49	8.2	Nd		+	+					
Gonen et al. [45]	2017	200	0	0	+	+						+
Jackson et al. [46]	2017	11	0	0	+	+						
Krishnan et al. [47]	2017	18	Nd	0	+	+	+			−	−	
Labib et al. [48]	2017	39	7.7	Nd	+	+						
Oertel & Burkhardt [51]	2017	16	0	0	=	=	+	+		=		+
Iyer & Chaichana [57]	2018	14	0	Nd	+	+	+					
Khalessi et al. [58]	2018	18	0	Nd	+	+	+	+		=		+
Mampre et al. [60]	2018	15	0	Nd	+	+						
Bakhsheshian et al. [63]	2019	25	4	0	+	+						
Garneau et al. [16]	2019	6	0	0	+	+		+		−	−	+
Herlan et al. [64]	2019	6	NA	Nd	+	=		+		=		
Kwan et al. [65]	2019	10	0	Nd	+	+	+	+	+			+
Muhammad et al. [7]	2019	8	0	0	+	+		+		−		
Murai et al. [67]	2019	22	0	18.2%	+			+	+			
Smith et al. [69]	2019	11	0	36.4%	=	=		+	+			+
Ahmad et al. [9]	2020	22	0	Nd	+	+	+	+			=	
Ariffin et al. [15]	2020	69	5.8	Nd	+	+	+	+	+	+		+
Baron et al. [10]	2020	28	3.6	Nd	+	+						
Burkhardt et al. [71]	2020	34	Nd	17.6%	=/+	=/+				−		
Chen et al. [73]	2020	39	30.8	Nd		=		+			=	+
Eichberg et al. [30]	2020	56	8.9	Nd	=	=		=				
Hafez et al. [23]	2020	100	NA	Nd	=	+			+			
Pafitanis et al. [83]	2020	15	NA	Nd	=	=						
Roethe et al. [85]	2020	29	6.9	3.4%	−	=	=	+			=	
Siller et al. [5]	2020	60	0	0	−	=	=	+	+	−	=	+
Teo et al. [29]	2020	8	0	Nd	=/+	=/+			=/+			
Visocchi et al. [89]	2020	6	0	Nd	+	+						
Amoo et al. [90]	2021	18	0	0	=			+	+			+
Muscas et al. [27]	2021	14	0	57.1%	+	+		+				+
Muto et al. [93]	2021	5	0	0	=	=						
Rösler et al. [26]	2021	39	0	69.2%	+			+		−		
Rotermund et al. [95]	2021	296	0	0	+	+	+	+			=	
Shimizu et al. [96]	2021	14	0	0		+		+				
Yoon et al. [25]	2021	11	9.1	18.2%	=	+		+	+		−	

+, superior compared to OM; =, similar compared to OM; −, inferior compared to OM; NA, not applicable; Nd, not available.
